# Influence of *Tagetes patula* and *Viola tricolor* on survival of *Staphylococcus aureus* ATCC 25923

**DOI:** 10.1016/j.heliyon.2022.e11777

**Published:** 2022-11-21

**Authors:** Izabela Steinka, Jadwiga Stankiewicz, Anita Kukułowicz, Aleksandra Wilczyńska

**Affiliations:** Gdynia Maritime University, Faculty of Management and Quality Science, Department of Quality Management, 81-225 Gdynia, Morska 81-87, Poland

**Keywords:** *S. aureus* ATTC 25923, *Tagetes patula*, *Viola tricolor*, Biostatic

## Abstract

So far, no studies have assessed the antibacterial properties of macerates of flower petals intended for human consumption. Previous studies have focused on the role of extracted flower components in inhibiting bacterial growth, not considering the petal tissue as a mixture of different components. The aim of this study was to assess the inhibitory effect of unpreserved macerates and juices derived from edible flower petals of *Viola tricolor* and *T. patula* on the population of *Staphylococcus aureus* ATCC 25923. Evaluation of the biostatic properties of flowers was carried out in two stages: using the Baird-Parker RPF culture method and the disc diffusion method. The reduction in the number of staphylococci by the macerates of the petals and their mixtures did not exceed 11% of the inoculum value. A low degree of inhibition of *S. aureus* ATCC 25923was found with *T. patula* macerate and sap in studies using both methods. The diffusion disc method in the study revealed the synergistic effect of the petals of both species on *S. aureus* ATCC25923 cells.

## Highlights

• The reduction in staphylococci number by macerates of the petals and mixtures didn’t exceed 11% of the inoculum value.
• The results obtained by the disc diffusion method showed a weak biostatic effect of both individual flowers and mixtures.

## 1 Introduction

The consumption of edible flowers is a contemporary dietary trend (Benvenuti and Mazzonchini, 2021; Kumari et al., 2021). Studies on this subject are focused on their health-promoting properties, indicating the anti-inflammatory, anti-cancer, neuroprotective and anti-aging properties of edible flowers (Chitrakar et al., 2019; Kalemba-Drożdż and Cierniak, 2019; Mlcek et al., 2021; Navarro-Gonzales et al., 2015; Pires et al., 2018; Stefaniak and Grzeszczuk, 2019). Recently, the toxicological aspects related to the consumption of flowers have also been investigated (Drava et al., 2020; Kalemba-Drożdż, 2019; Lu et al., 2016; Guiné et al., 2021). Some studies have focused on consumer acceptance and preferences related to the organoleptic characteristics of flowers as well as consumer knowledge about the preservation of flowers (Guiné et al., 2021; Simoni et al., 2018). However, data regarding the changes in the microbiological quality of flowers during postharvest storage are scarce, raising the question of the safety of these raw materials (Wilczyńska et al., 2021; Marchioni et al., 2020). The flowers of rose, bitter orange, violets, nasturtiums, pansies, and marigolds have been known and used in many regional cuisines for many years (Benvenuti et al., 2016; Kaleba-Drożdż and Cierniak, 2013; Kalemba-Drożdż, 2016; Kumari et al., 2021; Pires et al., 2018).

Among the many types of known edible flowers, pansies (*Viola tricolor*), marigolds (*Tagetes patula*) and roses (*Rosa* spp.) are listed as the most common ingredients in dishes (Kaleba-Drożdż and Cierniak, 2013). The health-promoting or healing properties of flowers of the genera *Viola* spp. or *Tagetes* spp. are known and cited extensively in the literature, with emphasis on the antioxidant, immunosuppressive, neuroprotective and antimicrobial properties of selected ingredients (Faizi et al., 2008; Fernandes et al., 2019; Gautam and Kumar, 2017; Jain et al., 2012; Jha et al., 2012; Khoshkam et al., 2016; Latifian et al., 2021; Moliner et al., 2018; Prabawati et al., 2021; Yousuf et al., 2021).

However, there are no studies on the antibacterial properties of macerates of flower petals intended for direct consumption. Available data are mainly related to the role of the extracted flower components in inhibiting bacterial growth, not considering the petal tissue as a mixture of different components. In this context, the aim of this study was to evaluate the inhibitory effect of unpreserved macerates and sap derived from edible flower petals of *V. tricolor* and *T. patula* on *Staphylococcus aureus* ATCC 25923 populations.

## 2 Materials and methods

### 2.1 Plant material

We used flowers of the species *V. tricolor* and *T*. *petula* from the “Lawenda” (Horticultural Farm, Gdansk, Poland). In Lawenda, you can find over 200 different products - these are vast varieties of edible herbs and flowers**.** The flowers were harvested in June 2021, and the study was performed in four series in the laboratory of the Gdynia Maritime University.

### 2.2 Bacterial strain and culture conditions

The bacterial strain *S. aureus* ATCC 25923 was obtained from the Merck Collection (Poland), and a broth culture of *S. aureus* ATCC 25923 was prepared. The bacterial culture was obtained by reviving the lyophilisates in the liquid nutrient broth after 24–48 h of incubation at 37 °C. The inoculum was prepared on Baird-Parker RPF medium (bioMerieaux, Poland). The obtained broth culture was poured into 8- and 5-mL test tubes.

### 2.3 Preparation of plant material and determination of microbial count

To determine the *Staphylococcus* count, 2 and 5 g of flowers were washed three times in sterile distilled water by transferring them from one container to the next. Subsequently, the flower petals were homogenised with a Stomacher Lab-Blender 400 (Seward, Worthing, UK) homogeniser to prepare the macerates. Biostatic activity was tested by the disc-diffusion method, and the petals were pressurised. Sap was pressed from 10 ± 1 g of flower petals. Flower petals adhered to the discs and were covered with aluminium foil; the layers prepared in this way were subjected to a pressure of 156 kPa (Zaidan et al. (2005) modified by Steinka and Voelkner). Flower macerates were added to broth cultures of *S. aureus* ATCC 25923, with initial population of 5.18–6.64 log CFU/mL.

### 2.4 Assessment of biostatic activity

Evaluation of the biostatic properties of flowers was carried out in two stages:

Stage I - evaluation of the reduction of the number of *S. aureus* by the culture method in Baird-Parker RPF medium.

Stage II - assessment of growth inhibition by the disc-diffusion method.

#### 2.4.1 Culture-based method

The macerated flower petals, we transferred 2 g into 8 mL of *S. aureus* broth and 5 g into 5 mL of broth. After a reaction period of 30 and 120 min, 1 mL of the bacterial culture was collected, diluted and spread on Baird-Parker RPF medium, followed by incubation for 48 h at 37 °C. After incubation, the number of *S. aureus* ATCC25923 was determined in accordance with the methodology contained in PN-EN ISO 6888-1, 2001. The R staphylococcal number reduction index was determined to assess the reduction in the number of bacteria, using the following equation:

R = K − N_30–120_ ∗ 100%,

where

K-initial population of *S. aureus* ATCC25923,
N_30_- number of *S. aureus* ATCC25923 after 30 min of incubation,
N_120_ - number of *S. aureus* ATCC25923 after 120 min of incubation.

#### 2.4.2 Disc diffusion method

The antibacterial properties against *S. aureus* ATCC2523 (Merck, Poland) were determined by the disc diffusion technique. This method is based on the principle that the antimicrobial components of petals in the discs will diffuse into the media and inhibit the growth of sensitive organisms, thereby creating a zone around the disc. For this, 1 mL of *S. aureus ATTC* 25923 culture with a known inoculum density was spread on a plate containing Agar Baird-Parker RPF (bioMerieux, Poland). A suspension was prepared from the 24-hour bacterial culture; inoculum density was 5.97 log CFU/mL. The petals of each species of flowers and their mixtures were subjected to pressure, soaking their juice in paper discs with a diameter of 10 mm.

**Figure 1:**
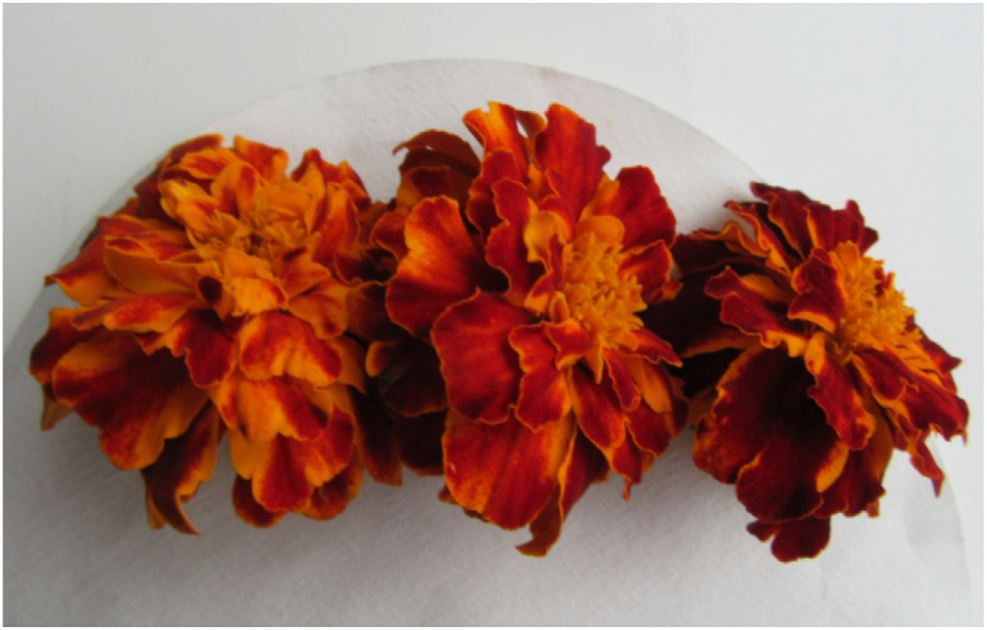
Variant A – a layer of marigolds adjacent to the disc.

**Figure 2:**
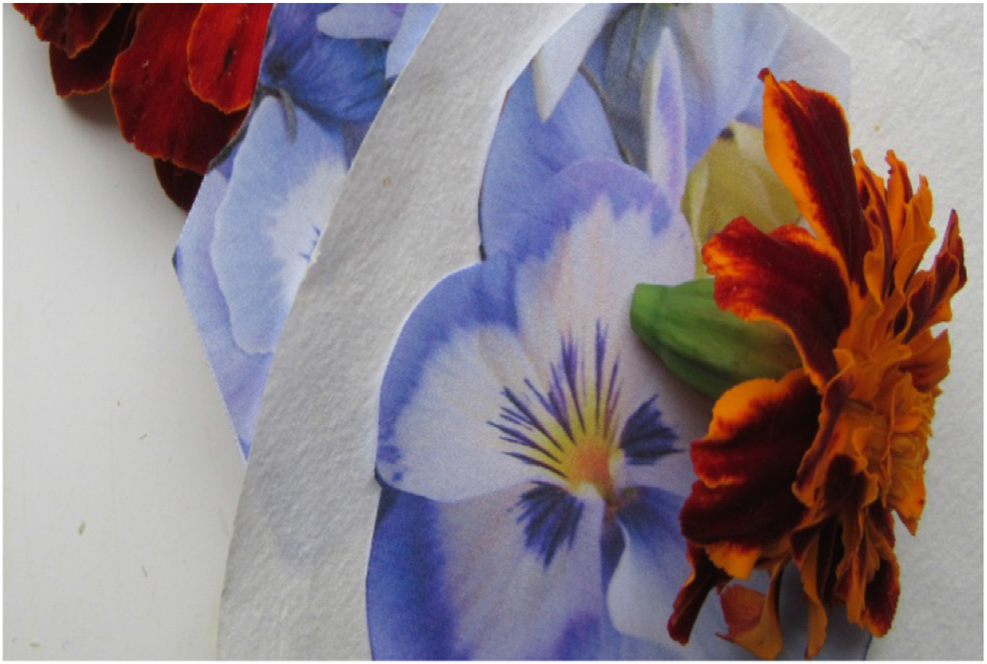
Variant C – a layer of pansies adjacent to the disc. The outer layer consist of marigolds.

The discs were soaked with the juice of marigold (A), pansy (B), and a mixture of AB/BA (C) and BA/AB (D), where AB/BA were present in as follows (from the outer to the inner part): aluminium foil-marigold/pansy-disc-pansy/marigold-aluminium foil. For BA/AB, the order was aluminium foil-pansy/marigold-disc-marigold/pansy-aluminium foil (Figures 1 and 2).

After drying the plate with the bacteria, discs with a diameter of 10 mm, soaked in the juice of the petals, were placed onto the plate. Subsequently, using sterile tweezers, discs were subjected to natural juice of marigolds and pansies and a mix of both; pure discs were used as control. Each disc was gently pressed, ensuring even contact with the medium. Plates were incubated under aerobic conditions for 24–48 h at 37 °C. Aft er incubation, a calliper gauge was used to determine the width of the microbial growth inhibition zone. Results are given as diameter of the zone growth inhibition in mm (Dasgupta et al., 2012). The degree of the sensitivity of staphylococci to the action of flowers was determined based on the size of the inhibition zone (CLSI, 2011).

### 2.5 Statistical analysis

We used the StataStatistical Software Release 15. StataCorp 2017 and Microsoft Excel – Microsoft Office Professional Office – Excel 2010 for data analysis. The results of four series of tests obtained in the first stage of the experiment were analysed using the paired t-test (mean comparison test) to determine whether the mean of a dependent variable is the same in the related groups. The results are expressed as mean and standard deviation. Correlation functions were calculated using the Statware office Excel 2010 software. Correlation equations were determined between the number of *S. aureus* during interaction with flower macerates and the time of co-incubation of bacteria with petals. The coefficients of determination of the equations illustrating the changes in the number of staphylococci, depending on the reaction time and the concentration of flower macerates, were determined.

## 3 Results and discussion

Evaluation of the number of *S. aureus* after interaction with *Viola* and *Tagetes* macerates and their mixtures showed that staphylococcal reduction and staphylococcal inhibition depended on the reaction time (Table 1). After 30 min of incubation, staphylococcal reduction by *Tagetes* was low (3.1%). The highest degree of reduction was observed at this time after the interaction of the bacteria with a 5% mixture of both flowers (*V. tricolor* and *T. patula*).

**Table 1:** Changes in the number of *S. aureus* ATCC 25923 during interaction with flower macerates and their mixture [log CFU/g].

Kind of samples	Macerate concentration [%]	Mean Standard Deviations	R _30_ [%]	Mean Standard Deviations	R_120_ [%]
*Tagetes patula*	2	5.44 ± 0.78	8.9	5.33 ± 0.70	10.8
*Tagetes patula*	5	5.19 ± 0.77	3.1	5.16 ± 0.80	3.6
*Viola tricolor*	2	5.52 ± 0.40	7.6	4.74 ± 0.63	20.7∗
*Viola tricolor*	5	5.49 ± 0.62	8.1	6.13 ± 0.35	+2.6∗∗
*Tagetes patula + Viola tricolor*	2	5.78 ± 0.58	3.2	5.79 ± 1.07	3.1
*Tagetes patula + Viola tricolor*	5	5.37 ± 0.73	10.1	5.73 ± 0.51	4.1

∗ significant differences (time),

∗∗ significant differences (concentration), R_30_ - value of the change in number *S. aureus* after 30 min, R_120_ - value of the change in the number of *S. aureus* after 120 min.

Both time and concentration determined the behaviour of *S. aureus* during exposure to *Viola* macerate. A statistically significant difference was found for the 120 min incubation time of staphylococci with 2% *Viola* macerate, (mean = 1.23; SD = 0.57; *P* = 0.023). The concentration of the *Viola* macerate used in the experiment was statistically significant, with *P* = 0.008 after 120 min of incubation. For *Viola*, 97.6% of the observed reduction in the number of *S. aureus* was determined by the incubation time (R^2^ = 0.976) (Table 2). We observed no statistically significant difference for the effect of both *T. patula* concentrations on the number of *S. aureus* (*P*_(T > t2)_ = 0.392; *P*_(T > t5)_ = 0.485). Similarly, no significant difference was found regarding the effect of interaction time on staphylococcal count reduction (*P*_(T > t120)_ = 0.16; *P*_(T > t 120)_ = 0.04).

**Table 2:** Effect of interaction time of 2% and 5% macerates on survival rate of *S. aureus* ATCC 25923.

Type of flowers	Concentration of macerates [%]	Correlation equations	Coefficient of determination R^2^
*Tagetes patula*	2	y = −0.32x + 6.22	0.874
*Tagetes patula*	5	y = −0.405x + 6.25	0.777
*Viola tricolor*	2	y = −0.615x + 6.64	0.976
*Viola tricolor*	5	y = 0.56x^2^ − 2.16x + 7.57	1
*Tagetes patula + Viola tricolor*	2	y = −0.09x + 6.026	0.708
*Tagetes patula + Viola tricolor*	5	y = −0.12x + 5.93 y = 0.48x^2^ − 2.04x + 7.53	0.157 1

x - interaction time between *S. aureus* and flower macerate.

The variability of the number of staphylococci under the influence of flowers could be described by a linear equation (Table 2). A 2.5-fold reduction in population size after interaction with the macerates was observed for the 5% flower mixture compared to the 2% mixture. Based on our results, factors other than time influenced the bacterial behaviour; only 15% of the change in number was determined by the total duration of the interaction between the macerate and the bacteria.

After 30 and 120 min of contact between the staphylococci and the macerates, we observed interactions between the components of both flowers. These interactions did not result in any further inhibition of the bacteria at the end of the experiment. The adjustment of the obtained data to the real conditions is illustrated by the second-degree polynomial equation (Table 2).

There is no information in the literature on the reduction in the number of *S. aureus* under the influence of macerates of edible flowers. The obtained data can be roughly compared with the results obtained for cold-water solutions of mixtures containing flowers. Evaluation of the biostatic properties of the aqueous solution of the mixture of holly and rose petals in interaction with *S. aureus* ATCC 25923 showed that the reduction in the number of staphylococci after 120 min was 8.2%. The observed decrease in the number of *S. aureus* was, on average, 0.5 log CFU/mL (Steinka, 2012, 2020). In the case of the multicomponent mixture of hibiscus and rosehip petals, the reduction was only 5.9% with a 0.5 McFarland inoculum. However, this mixture additionally contained cinnamon, cloves, dried apples, cherries and rosehips and orange peel in undefined proportions, suggesting that the components of the mixture could stimulate rather than inhibit the growth of staphylococcal cells. On the other hand, a high degree of inhibition of the *S. aureus* population was obtained when using an aqueous solution of *Camelia sinensis* with the addition of cornflower and bergamot oil, reaching a reduction of 83.9%.

The recorded decrease in the number of staphylococci was probably the result of the synergy of the antibacterial components of tea as well as the oil fraction of bergamot and cornflower flowers (Steinka, 2012, 2020). To date, the biostatic properties of edible flower macerates have not been investigated with the use of culture methods.

Our research shows that in the first phase of the interaction (30 min), the number of staphylococci decreased on average by 3.1–8.9% for both species of flowers, followed by 20% in the subsequent phase for *V. tricolor.* Instead of inhibition, a slight increase in the number of bacteria was also observed with the use of a higher concentration of *Viola* macerates, indicating the presence of *Viola* petal components in the broth medium during prolonged interaction (Table 1).

Based on the results of the disc diffusion method, there was a weak and moderate biostatic effect of both species and their mixtures (Table 3). The juice obtained during pressure maceration of the petals caused an inhibition zone with an average diameter of 8 mm for *Viola* and 14 mm for *Tagetes*. The disks were soaked with the mixture in two variants, when *Viola* (C) (Figure 2) or *Tagetes* (D) adhered to the surface of the disks during pressing. The observed changes in inhibition zones indicate a more effective biostatic effect of *Viola* juice directly adjacent to the disc (13.1 mm) compared to that observed for variant D (Table 3). The zone diameters obtained by us were smaller compared to those obtained for the extracts of other edible flowers. For example, Evidente et al. (2004) assessed the effect of amaryllis extracts and obtained inhibition zones from 17 to 22 mm, indicating a high sensitivity of *S. aureus.*

**Table 3:** *S. aureus* ATCC 25923 inhibition zones with macerates of marigold, pansies and their mixtures.

Kind of samples	Zone Diameter [mm] Mean value, SD	Zone diameter – strain resistance according to CLSI	Degree of sensitivity for variants A,B,C,D ID
A	11.4 ± 0.83	≤14 mm-resistant	Low
B	10.8 ± 0.69	≤14 mm- resistant	Low
C	13.1 ± 0.72	≤14 mm- resistant	Low
D	11.9 ± 1.24	≤14 mm- resistant	Low

A-marigold, B-pansy, C - petals arranged on the disc in the order AB /BA, D-petals arranged on the disc in the order BA/AB.

It should be noted that these differences may have resulted from the use of substances extracted from flower petals and not their macerates. In this study, direct penetration of the surface of staphylococcal cells by the juice soaked in the disc was obtained. Also, *S. aureus* ATCC 25923 showed low sensitivity to the action of *Viola*, *Tagetes* and mixtures of both flowers (Table 3).

There are no data in the literature on the biostatic properties of edible flower macerates or their juices, assessed by the diffusion disc method. Most studies focused on water extracts, ethanol extracts, methanol-water extracts or extracts in which the solvents are hexane or ethyl acetate. There are no unequivocal results regarding the biostatic properties of *Viola* in studies conducted with the use of extracts of various species of this genus (Gautam and Kumar, 2017; Muhammad et al., 2012)*.* Studies using the species *Viola betonicifolia* reported no inhibition of *S. aureus* for both aqueous flower extracts and methanol solutions or with ethyl acetate or hexane. Antibacterial activity was assessed using the disc diffusion method. According to some authors, the antimicrobial activity of the aqueous *Viola odorata* extract assessed in relation to *S. aureus* is as high as 41.8 ± 2.4%. Also, a high sensitivity of *S. aureus* to the aqueous extract was noted. The maximum inhibition of staphylococci was obtained using a 1-μg/mL solution of *Viola* (Gautam and Kumar, 2017).

When using the oil obtained from *Viola partinii*, the inhibition zone of *S. aureus* was 6.62 mm at a concentration of 0.250 mg/mL (Yousuf et al., 2021). Khoshkam et al. (2016) obtained an inhibition zone of 10 mm using a butanol extract of proteins from *V. tricolor* flowers. This degree of biostatic activity of *Viola* extracts obtained by the disc diffusion method is similar to that obtained in our experiment. Jha et al. (2012) indicate that water extracts from flowers of the genus *Viola spp.* do not show any ability to inhibit *Staphylococcus aureus.* This confirms our theory that it is necessary to study the effects of all petal components in unpreserved macerated tissue or sap. The use of a methanol-water extract of macerated flowers and leaves derived from *T. patula* resulted in an inhibition zone comparable to that observed in our experiment (Latifian et al., 2021). The high concentration of methanolic flower extract caused an inhibition zone of up to 12 mm, whereas plant leaf extracts resulted in a zone of up to 10 mm. Faizi et al. (2008) showed that the methanol-water extract of *T. patula* flowers caused the formation of an inhibition zone with a diameter of 10–14 mm, whereas the patuletin component extracted from these flowers produced a zone of 8–23 mm. These authors concluded that *T. patula* flowers constitute a good mixture of components with antimicrobial properties. Comparable results were obtained by Jain et al. (2012), who studied water extracts of *Tagetes erecta* and *T. patula* flowers. The authors observed a high antimicrobial activity against *S. aureus*, with an inhibition zone of 26 mm. The diameters of the staphylococcal inhibition zones obtained with the use *Tagetes* petal juice obtained in our experiment were about 54% smaller compared to those obtained by Jain et al. (2012).

## 4 Conclusions

The reduction of staphylococci number by macerates of the petals and their mixtures did not exceed 11% of the initial population value. Low inhibition of *S. aureus* ATCC 25923 was found for the macerate and *T. patula* sap, using both methods. The diffusion disc method revealed the synergistic effect of the petals of both species on *S. aureus* cells ATCC25923. The contact of the *Viola* sap with the outer coatings of the staphylococcal cells in the mixture (variant C) indicates easier penetration and a higher degree of inhibition of bacteria compared to *Tagetes*. Our results lead us to infer that salads containing a mixture of *Viola* and *Tagetes* flower petals show higher biostatic activity in relation to staphylococcal cells compared to those only containing the flowers of one species.

## Declarations

### Author contribution statement

Izabela Steinka: Conceived and designed the experiments; Performed the experiments; Analyzed and interpreted the data; Wrote the paper.

Jadwiga Stankiewicz; Anita Maria Kukułowicz,; Aleksandra Wilczyńska: Contributed reagents, materials, analysis tools or data.

### Funding statement

This work was supported by UMG Departments for Research activities under project number [WZNJ/2022/PZ/01].

### Data availability statement

Data will be made available on request.

### Declaration of interest’s statement

The authors declare no conflict of interest.

### Additional information

No additional information is available for this paper.
